# Physician assistants working in primary care in Canada: Findings from a national survey

**DOI:** 10.1177/08404704251347908

**Published:** 2025-06-12

**Authors:** Kristen Burrows, Leslie Nickell, Paul Krueger

**Affiliations:** 1 62703 McMaster University, Hamilton, Ontario, Canada.; 2 University of Toronto, Toronto, Ontario, Canada.

## Abstract

Physician Assistants (PAs) are increasingly recognized as part of the solution to addressing Canada’s primary care shortage. This study reports findings from a national survey of 386 Canadian PAs with primary care experience. Respondents described delivering a broad scope of care, including direct patient management, teaching, mentorship, and quality improvement across settings such as elderly care, mental health, Indigenous health, refugee health, and rural communities. Most PAs reported high confidence in core competencies and effective integration into interprofessional teams. Despite this, systemic barriers persist including inadequate funding, role ambiguity, and resistance from other providers. Many PAs (71%) expressed job satisfaction, and 75% would recommend primary care practice. The study highlights opportunities to improve PA utilization and access to care through policy reform, better funding models, and expanded educational supports. These insights are valuable for policy-makers, administrators, and educators aiming to strengthen primary care delivery and PA role optimization.

## Introduction

In the face of the critical primary care crisis in Canada, with more than 6.5 million Canadians lacking a primary care provider or family physician,^1,2^ the integration of Physician Assistants (PAs) into primary care settings has emerged as a significant development aimed at enhancing the accessibility and efficiency of family medicine services.^3-5^ PAs are uniquely positioned to play a pivotal role in helping to mitigate shortages of family physicians by extending the reach of existing primary care physicians and improving patient access and outcomes.^5-8^

PAs work in collaboration with physicians to meet the healthcare needs of a specified patient population. The specific responsibilities of a PA are determined through discussions with their supervising physician, considering the PA’s training, experience, and professional strengths, as well as the preferences of the healthcare team, the needs of the patients, and the level of trust established between the physician and the PA.^9,10^ In primary care settings, the role of a PA extends physician services through direct patient care that includes history taking, physical examination, ordering of diagnostic tests, performing office-based procedures, providing prescriptions and other treatment recommendations, counselling, and referrals.

Previous Canadian research has demonstrated the benefits and challenges of PA integration into primary care settings,^11^ physician satisfaction with PA role integration,^12,13^ and explored patient experiences,^14^ yet the full contributions of PA roles in primary care have not been elucidated from the providers themselves. The purpose of this study was to delve deeper into PAs’ experiences and confidence while working in diverse primary care settings, including care of the elderly and long-term care, mental health, refugee health, Indigenous health, substance use health, and providing primary care in remote, rural, or northern communities. Additionally, the survey collected data on PAs’ perceptions of their ability to perform effectively in primary care and assessed how well their education programs prepared them for these roles.

By examining PA contributions to healthcare teams, this study addresses the scope of PA primary care responsibilities, the impact of their work on patient care, and the potential barriers and facilitators to their full integration into the healthcare system. The evolution of the PA profession in Canada, influenced by both policy changes and clinical needs, highlights the adaptive nature of this role and its importance in supporting primary healthcare services across diverse settings. Given the relative novelty of PAs in the Canadian medical landscape and the variability in their roles across different provinces, this investigation is timely and relevant. It aims to provide stakeholders, including healthcare providers, policy-makers, and educators, with a comprehensive understanding of how PAs can be effectively utilized to enhance the delivery of primary care. Through this exploration, the manuscript will contribute to the ongoing discourse on optimizing healthcare workforce strategies to ensure that all Canadians have access to high-quality primary care.

## Methods

### Questionnaire development

The questions were developed after a review of the literature to collect information about PAs’ experiences and confidence working in primary care settings with diverse populations including care of the elderly/long-term care, mental health, refugee health, Indigenous health, substance use health, and providing primary care in remote/rural/northern communities. The research team met frequently to refine the questions, considering issues of reliability, validity, readability, grammar, and appropriateness. The questionnaire was then pretested on a group that included content experts, a research methodologist, and potential respondents. The questionnaire was revised based on the pretesting and then formatted into an online web-based survey using QualtricsXM. The final draft of the survey was pilot tested and minor revisions were made based on the pilot testing. The final questionnaire contained seven sections designed to collect information on the following: PA program and work in primary care; primary care practice characteristics; self-assessment of their ability to perform in primary care; confidence working with diverse primary care communities/populations; demographic information; and general comments. The questionnaire can be obtained by contacting the corresponding author.

### Participant recruitment

The “Canadian Survey of Physician Assistants Working in Primary Care” was open to any Canadian (CCPA) or United States (US) (PA-C) certified PA working in Canada. The goal was to recruit all PAs who had ever worked in primary care in Canada since graduating from their respective PA programs. Participant recruitment was multifaceted and included study recruitment through the Canadian Association of Physician Assistants (CAPA) and through the existing PA education programs at the University of Manitoba, University of Toronto, and McMaster University (since this study was completed, new programs have been launched at the University of Saskatchewan, Dalhousie University, and the University of Calgary). Recruitment information was distributed by e-mail and posted links on respective social media sites.

### Survey implementation

A modified Dillman approach was used to maximize response rates while allowing flexibility in delivery mode and survey timing. The Dillman approach employs a structured series of participant contacts (such as personalized invitations, reminders, and follow-ups) to enhance participation.^15^ The CAPA and all three Canadian PA Programs sent an initial e-mail and three follow-up weekly reminders to their respective e-mail distribution lists. They all also posted a link to the survey on their respective Facebook pages.

### Analysis

The data were exported from QualtricsXM as an SPSS file for analysis. The questionnaire contained several questions with five-point Likert scales. Prior to analysis, the research team reached consensus on the most appropriate way to recode the categorical response data. For example, the five-point Likert scale to rate PA’s confidence (not at all confident, not very confident, neutral, fairly confident, and very confident) managing patients in/from various primary care communities/populations was dichotomized into fairly confident + very confident vs all other responses combined. Descriptive statistics were calculated for all variables, including frequency counts, and percentages for categorical variables, or means and standard deviations for continuous variables.

## Results

### Response rate

The actual number of PAs working in Canada is unknown and our best estimate based on available Canadian data at the time of the survey (registries, CAPA members) is 1,100. Assuming this number is accurate, our overall response rate for this survey was 503 (46%).

Exactly 503 PAs began the survey and responded to the first 3 questions (year graduated from PA program; which PA program they graduated from; and whether or not they have ever worked in Primary Care since graduating). Of the 503, 117 (23.3%) reported never having worked in primary care as a PA and were skipped to the end of the survey. The remaining 386 (76.7%) who reported ever working in primary care as a PA are the focus of this paper.

### Demographic and practice characteristics

Of the 386 PAs who reported ever working in primary care, 58% (223) graduated from an Ontario training program (36% from the University of Toronto and 21% from McMaster University), 9% (33) from the University of Manitoba, and 22% (84) graduated from the Canadian Armed Forces PA program (pre-2021). US trained PAs accounted for 12% (47) and two respondents did not specify. Respondents included PAs who graduated between 1990 and 2023 and first started working in primary care between 1991 and 2023.

The mean age of 259 respondents was 39 years of age with a range from 24 to 66 years of age. Of 264 respondents, 32.2% identified as males and 54.9% as females with the remaining 12.9% reporting either agender, gender fluid, gender queer, non-binary, self-described, or preferred not to answer. Of those who responded (254), the majority identified their ethnic group as white (80.3%), followed by South Asian (6.7%), Chinese (5.1%), Black (3.1%), Southeast Asian (2.8%), Latino (2.4%), Filipino (1.9%), Arab (1.2%), Caribbean (1.2%), Oceania (1.2%), West Asian and Middle East (0.9), Japanese (0.4%), or Korean (0.4) with the remaining either specifying their own ethno-racial identity (2.0%). 18.9% (48/255) of those who responded indicated that they identified as First Nations, Metis, or Inuit. Of those who responded 14.7% (36/245) self-identified as a person with a disability and/or impairment.

Of 312 respondents, 242 (77.6%) reported that they *currently* worked as a PA in primary care. The reasons for the 70 who reported no longer working in primary care included: change of speciality (58.6%); insufficient salary (28.6%); parental or medical leave (12.9%); change of career (10.0%); recent location move (10.0%); no jobs available (7.1%), and various other reasons (15.7%).

Of 303 respondents, PAs reported working an average of 35.5 paid hours per week (median 40.0 hours) in primary care as a clinical PA with an additional 4.3 unpaid hours/week (median 2.0).

Of 312 respondents, 61.2% of PAs reported working clinically in primary care with paediatric populations (less than 18 years of age); 92.0% with adults ages 18-64, and 66.0% with adults 65 years of age or older. Table 1 summarizes additional practice characteristics, including locations and types of communities/populations served, non-clinical (i.e., not patient facing) responsibilities, and identified interprofessional teams.

**Table 1.** Practice characteristics of PAs who reported ever working clinically in primary care.

Characteristics	Number (%)
Location where PAs ever worked clinically in primary care (n = 335^a^)	
Canada^b^	
Ontario	189 (56.4)
Manitoba	32 (9.6)
Alberta	24 (7.2)
British Columbia	14 (4.2)
Nova Scotia	11 (3.3)
Quebec	8 (2.4)
Northwest Territories	7 (2.1)
Saskatchewan	3 (0.9)
Nunavut	3 (0.9)
New Brunswick	0 (0.0)
Newfoundland and Labrador	0 (0.0)
Prince Edward Island	0 (0.0)
Yukon	0 (0.0)
United States	
17 different states	39 (11.6)
Outside of North America	
3 locations	5 (1.5)
Communities/Populations PAs provided care for (n = 312)	
Mental health (outpatient) including counselling, therapy, or other management	211 (67.6)
Care of the elderly (clinic or community setting, excluding long-term care)	206 (66.0)
Substance use/Addictions health	145 (46.5)
Indigenous populations	101 (32.4)
Remote/Rural/Northern settings	95 (30.4)
Refugee health (those who were forced to leave their home country)	71 (22.8)
Long-term care	68 (21.8)
Other^c^	34 (10.9)
Non-clinical PA responsibilities in their primary care roles (n = 312)	
Teaching	166 (53.2)
Mentorship	124 (39.7)
Quality improvement	91 (29.2)
Research	54 (17.3)
Leadership^d^	44 (14.1)
Other activities^e^	20 (6.4)
None (patient care only)	77 (24.7)
Professions PAs collaborated with in their clinical role in primary care (n = 249)	
Nursing (includes RN, RPN, and NP)	209 (83.9)
Pharmacy	186 (74.7)
Social work	157 (63.1)
Medical learners (PA or MD)	154 (61.8)
Physiotherapy	118 (47.4)
Dietitian/Nutritionist	108 (43.4)
Occupational therapy	88 (35.3)
Resident or fellow	79 (31.7)
Respiratory therapy	55 (22.1)
Speech language pathology	44 (17.7)
Spiritual care	30 (12.0)
Recreational therapy	24 (9.6)
Midwives	22 (8.8)
Child life specialist	11 (4.4)
Other^f^	37 (14.9)

^a^31 PAs reported working in multiple locations (ranging from 2 to 5).

^b^Includes Canadian Armed Forces PAs employed in base or primary care settings.

^c^Included CAF Primary Care, supporting marginally housed patients, patients experiencing homelessness, maternal/newborn care, Mennonite population, migrant workers, occupational medicine, sports medicine, transgender care, urgent care, or walk-in clinics.

^d^Included such things as: coordinator, manager, team lead, supervisor, department head, and preceptor.

^e^Included such things as: advisor, committee member, compliance, counselling, health & safety, interdisciplinary rounds, and participating on working groups.

^f^Included addiction counsellors, case management, chiropodist, dentist, health promotion, peer support, kinesiologists, paramedics, psychiatrist, psychologist, radiology, public heath, and sports medicine.

Of 307 respondents, 249 (81.1%) reported that they collaborate with other professions in their clinical PA role in primary care (Table 1), and 18.9% (58/307) of respondents indicated they only work with a supervising physician (no other healthcare team members). Of the 249 who reported collaborating with other professions, 89.5% (221/247) agreed or strongly agreed that their clinical role in primary care was part of an interprofessional team, 71.2% (176/247) rated the effectiveness of *teamwork* in their interprofessional team as being very good or excellent, and 65.6% (162/247) rated the effectiveness of *collaboration* in their interprofessional team as being very good or excellent.

### Self-assessment of ability to perform in primary care

PAs were asked to rate themselves on the professions 12 Entrustable Professional Activities (EPAs) in their role working in primary care. The percentage of PAs who rated themselves as very good or excellent ranged from 70.5% to 87.7% (Table 2).

**Table 2.** PA self-assessment ratings of entrustable professional activities (n = 292).

Entrustable Professional Activities (EPAs)	Self-rating of ability in primary care as very good or excellent number (%)
**EPA 1:** Practices patient-focused, safe, ethical, professional, and culturally competent medical care across the healthcare continuum	256 (87.7)
**EPA 2:** Obtains histories and performs physical examinations, demonstrating the clinical judgement appropriate to the clinical situation	252 (86.3)
**EPA 3:** Formulates clinical questions and gathers required clinical evidence to advance patient care and communicates those results to the patient and medical team	249 (85.3)
**EPA 4:** Formulates and prioritizes comprehensive differential diagnoses	227 (77.7)
**EPA 5:** Develops and implements patient-centred, evidence-based treatment plans within the formalized physician, clinical team, and caregiver relationship	234 (80.1)
**EPA 6:** Accurately documents the clinical encounter incorporating the patient’s goals, caregiver goals, decision-making, and reports into the clinical record	241 (82.5)
**EPA 7:** Collaborates as a member of an interprofessional team in all aspects of patient care including transition of care responsibility	235 (80.5)
**EPA 8:** Recognizes a patient requiring immediate care, providing the appropriate management and seeking help as needed	249 (83.5)
**EPA 9:** Plans and performs procedures and therapies for the assessment and the medical management appropriate for general practice	228 (78.1)
**EPA 10:** Engages and educates patients on procedures, disease management, health promotion, wellness, and preventive medicine	239 (81.8)
**EPA 11:** Recognizes and advocates for the patient concerning cultural, community, and social needs in support of positive mental and physical wellness	212 (72.6)
**EPA 12:** Integrates continuing professional and patient quality improvement, life-long learning, and scholarship	206 (70.5)

When asked to rate their overall competency level working in primary care, 70.2% (205/292) rated themselves as very good or excellent.

PAs were asked to rate their confidence level managing patients in/from various primary care communities/populations. Ranked from high to low, the percentage of 287 PAs who reported being fairly or very confident in managing patients is as follows:

• 72.3% for mental health (outpatient) including counselling, therapy, or other management;
• 69.7% for care of the elderly (clinic or community setting, excluding long-term care);
• 54.0% for remote/rural/northern settings;
• 47.4% for Indigenous populations;
• 39.7% for substance use/addictions health;
• 36.6% for long-term care; and
• 30.0% for refugee health (those who were forced to leave their home country).

### Barriers and facilitators to PAs working in primary care

PAs were asked to rate the importance of potential barriers in limiting their ability to work to their full potential as a PAs in primary care and also to rate the importance of potential facilitators in helping them work to their full potential as a PAs in primary care (Table 3).

**Table 3.** Percent of PAs who rated barriers and facilitators as fairly or very important in limiting or helping them to work to their full potential as a PAs in primary care.

Potential barriers and facilitators	Fairly or very important number (%)
Potential barriers (n = 273)	
Funding	213 (78.0)
General misunderstanding of the role of PAs	197 (72.2)
Medical directives	160 (58.6)
Lack of role clarity	159 (58.2)
Resistance from other healthcare providers	148 (54.2)
Personal factors (such as family demands, childcare, and personal health)	145 (53.1)
Poor relationship with supervising physician	140 (51.3)
Lack of knowledge	131 (48.0)
Patient willingness to be seen by a PA	129 (47.3)
Hospital policies limiting PA visits to rostered patients	118 (43.2)
Lack of physical space to see your own patients	112 (41.0)
Multiple supervising physicians	112 (41.0)
Role overlap	100 (36.6)
Unionization	82 (30.0)
Lack of unionization	74 (27.1)
Potential facilitators (n = 269)	
Collaborative work environment	235 (87.4)
Degree of independence/autonomy in providing patient care	231 (85.9)
Focus on team-based care	226 (84.0)
Opportunities to interact with other professions (interprofessional care)	221 (82.2)

Exactly 69.8% (187/268) of PAs agreed or strongly agreed that in their most recent or current PA roles in primary care that they were working to their full potential and 71.2% (191/268) responded that overall, they were satisfied or very satisfied with their most recent or current PA roles in primary care. Exactly 75.4% (202/268) of PAs reported that they would recommend to other PAs that they should work in primary care.

## Discussion

The role of PAs in primary care across Canada has increasingly become a focal point for healthcare system enhancements, particularly in underserved and rural communities. Our survey, capturing responses from a substantial cohort of PAs, sheds light on the critical functions and challenges that define this profession within the primary care landscape.

Our data indicate that most PAs are actively engaged in diverse and crucial areas of patient care, including mental health, care of the elderly, and substance use health. With 92% of respondents involved in adult care and significant numbers contributing to paediatric and geriatric care, PAs are addressing healthcare needs and bridging gaps across all age groups. Furthermore, the breadth of responsibilities, including teaching, mentorship, and quality improvement, underscores the versatile and integral role PAs play in the healthcare ecosystem in Canada and internationally.^5,7,8,16,17^

The high percentage of PAs working in collaborative primary care environments (81.1%) with other healthcare professionals highlights the interprofessional nature of their roles.^18^ PAs in primary care most often collaborate with nursing, pharmacy, social work, physiotherapy, and occupational therapy. The strong self-reported competency levels across a wide range of EPAs reflect both the effectiveness of PA training programs and their professional adaptability. This has been previously echoed through employer and physician supervisor satisfaction.^12^ Self-reported role satisfaction of PAs working in primary care was 71.2%, which is consistent with general role satisfaction of Canadian family physicians (72%) and slightly below academic family medicine faculty (89.7%).^19^

A substantial portion of PAs cited barriers such as funding, role clarity, and resistance from other healthcare providers pointing to systemic issues that could impede the full utilization of PAs in primary care settings and reduces role satisfaction. The lack of permanent and equitable funding mechanisms (i.e., poor remuneration compared to acute care settings or pay inequity across similar health professional roles) aligned with broader health human resource priorities leads to instability and restricts optimal utilization of PAs. Misunderstanding and ambiguity about the PA role—including scope of practice, required supervision models, and “assistant” title—create uncertainty among other healthcare providers and patients, resulting in underutilization and resistance to role integration. Resistance from other healthcare providers, stemming from perceived role overlap, competition for resources, or role misunderstanding, further complicates integration efforts, though this typically diminishes as familiarity and experience with PAs increase. The barriers cited by study participants are not new and are consistently echoed in other research.^11,13,20^

### Recommendations

To effectively address these barriers and enhance the integration of PAs in primary care, several strategies are recommended. Sustainable funding models, specifically designed to support PA roles within interdisciplinary team-based care, are essential and should link compensation to indicators of collaborative performance and team cohesion.^21^ These models could include recognizing PAs as key family health team members, ensuring pay equity across advanced care provider roles, or allowing a supervising physician to bill for a portion of PA visits. Enhancing role clarity and normalization through public education,^22-24^ boosting interprofessional learning or collective training opportunities, and the adoption of modernized delegation frameworks (such as collaborative practice agreements instead of medical directives) will improve role flexibility, patient understanding, and team acceptance.^25^ PA training programs should expand to meet demand, continue to incorporate interprofessional competencies,^26^ collaborate with other healthcare training programs, integrate additional rural and underserved clinical training exposure, and build research and health management curriculum. Fostering interprofessional collaboration and leadership roles for PAs,^27^ alongside dissemination of evidence highlighting their positive impact on care quality, will further encourage acceptance and effective integration.

### Future directions

Ongoing research assessing the impact of PAs on patient outcomes, system efficiency, and healthcare economics will be critical in informing resource allocation and policy decisions.^5^ Such evidence will help to strategically address integration barriers, guiding health leaders in making informed, supportive policy reforms. Given variations in government funding models across Canada, future modelling needs to include setting-specific data and key funding details to fully understand the cost effectiveness and full potential of PA integration.^28^ Policy-makers should explicitly include PAs in national and provincial healthcare planning strategies, ensuring these plans reflect the evolving competencies and contributions of the PA profession within the evolving healthcare landscape.

### Limitations

As with all survey-based research, there are some limitations. Response rate challenges were primarily due to limited access to up-to-date or comprehensive contact lists for the Canadian PA population. Specifically, we were unable to send personalized e-mail invitations to PAs employed in Canada, and instead relied on generic survey links, which typically yield lower engagement. Despite this, the sample demographics and practice characteristics were broadly representative, as reflected in Tables 1 and 2, suggesting that the results are still informative. Future efforts could benefit from personalized outreach and access to complete contact databases to maximize participation and enhance generalizability.

This study benefited from several strengths, including the use of a comprehensive, rigorously developed questionnaire, informed by prior research and refined through pretesting and pilot testing. The sample size was relatively large, and the findings are consistent with those reported in the existing literature, enhancing the credibility of the results.

## Conclusion

PAs are making important contributions to primary care in Canada, delivering comprehensive, team-based care across a wide range of patient populations and clinical settings in collaboration with family physicians. Their high levels of self-reported competence and confidence, particularly in underserved areas such as elderly care, mental health, and rural communities, highlight their value as adaptable and skilled providers. Despite persistent systemic barriers—such as funding challenges and role ambiguity—PAs remain committed, with the majority expressing job satisfaction and a willingness to recommend primary care to peers. Optimizing PA integration through supportive policies, role clarity, and improved funding models presents a timely opportunity to strengthen primary care delivery and address Canada’s health human resource challenges.
